# Supplementary material for: New Copper(I) Complex with a Coumarin as Ligand with Antibacterial Activity against Flavobacterium psychrophilum
Source: Molecules. 2020 Jul 13;25(14):3183. doi: 10.3390/molecules25143183 (PMC7397040; doi:10.3390/molecules25143183)
Supplement: Supplementary file 1 [file molecules-25-03183-s001.pdf]

Supplementary Material

New copper(I) complex with natural product coumarin as ligand with antibacterial activity against *Flavobacterium psychrophilum*

Maialen Aldabaldetrecu [1], Mick Parra [2,3] , Sarita Soto [4], Pablo Arce [1], Mario Tello [3] , Juan Guerrero [1,*] and Brenda Modak [2,*]

[1]Laboratory of Coordination Compounds and Supramolecularity, Faculty of Chemistry and Biology, University of Santiago of Chile; maialen.aldabaldetrecu@usach.cl; pablo.arce@usach.cl, juan.guerrero@usach.cl

[2] Laboratory of Natural Products Chemistry, Centre of Aquatic Biotechnology, Faculty of Chemistry and Biology, University of Santiago of Chile; mick.parra@usach.cl; brenda.modak@usach.cl

[3] Laboratory of Bacterial Metagenomic, Centre of Aquatic Biotechnology. Faculty of Chemistry and Biology, University of Santiago of Chile; mick.parra@usach.cl; mario.tello@usach.cl

[4] Laboratory of Biochemistry and Oral Biology. Faculty of Odontology, University of Chile sarita.soto@usach.cl

* Correspondence: brenda.modak@usach.cl; Tel.: + 56-2-27181147; juan.guerrero@usach.cl; Tel.: +56-2-2781086

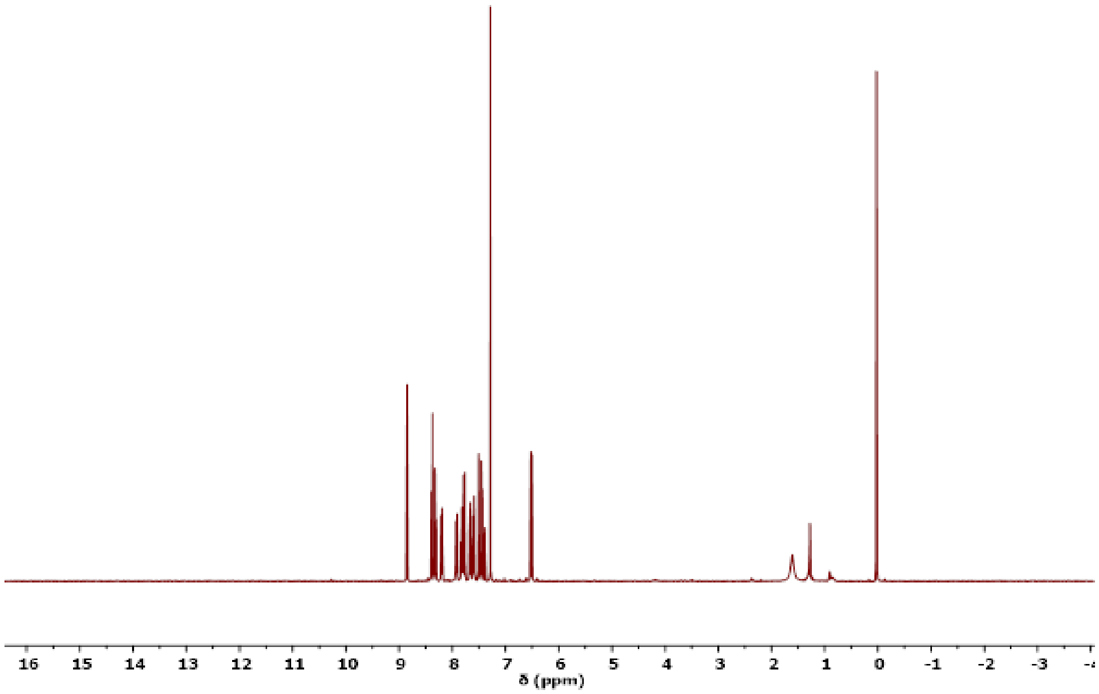

Figure S1. ^{1}H-NMR spectrum of **NN$_1$. in $CDCl_3$ at 300K**.

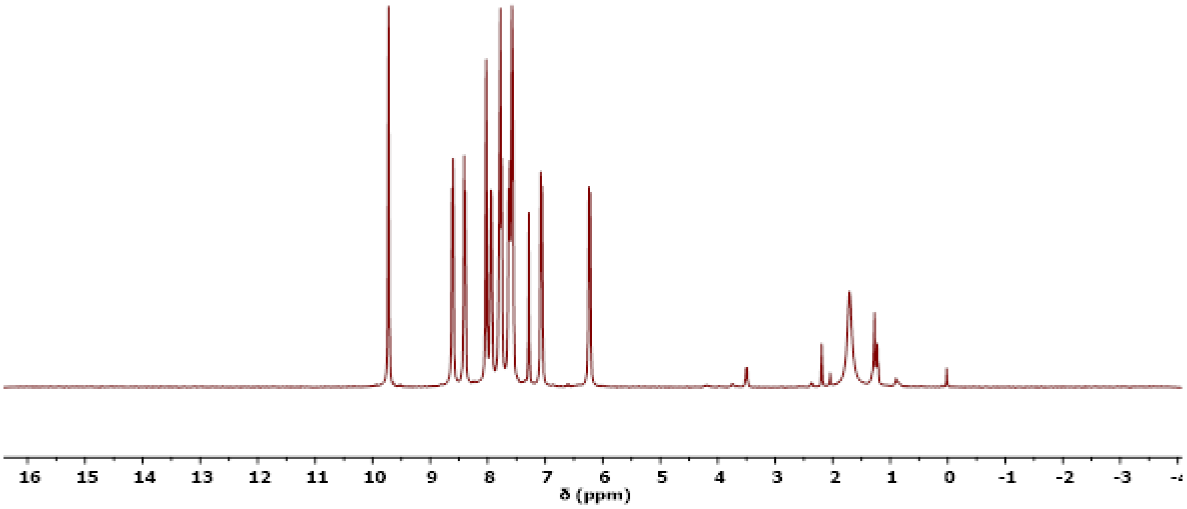

Figure S2. ^{1}H-RMN spectrum of **[Cu(NN$_1$)$_2$]ClO$_4$**. in **CDCl$_3$ at 300K**.

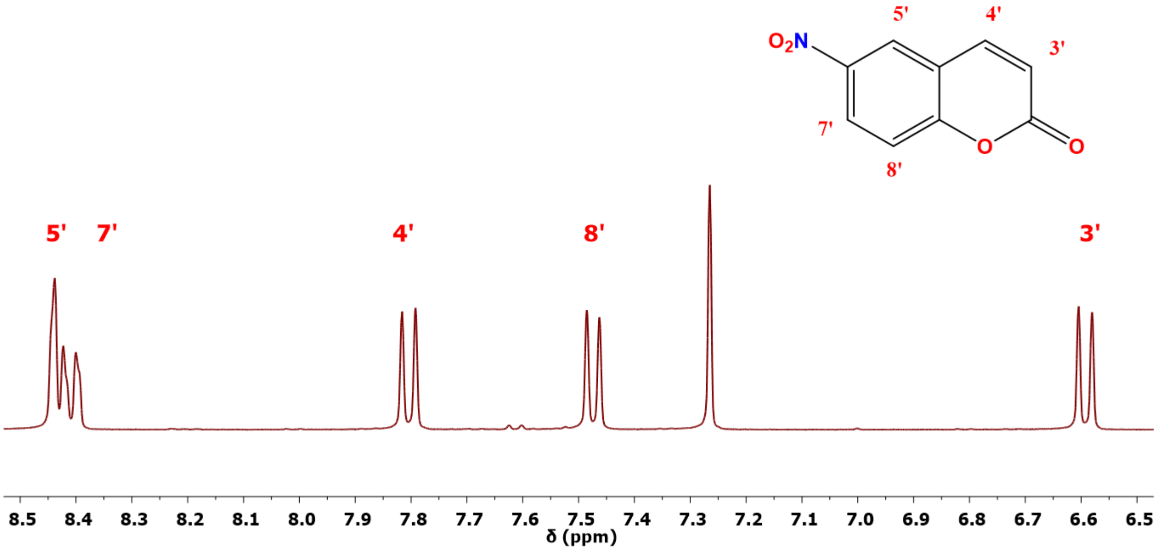

Figure S3. ^{1}H-NMR spectrum of **6′-nitrocoumarin in CDCl$_3$ at 300K**. Insert: Structure with atom numbering for proton assignments.

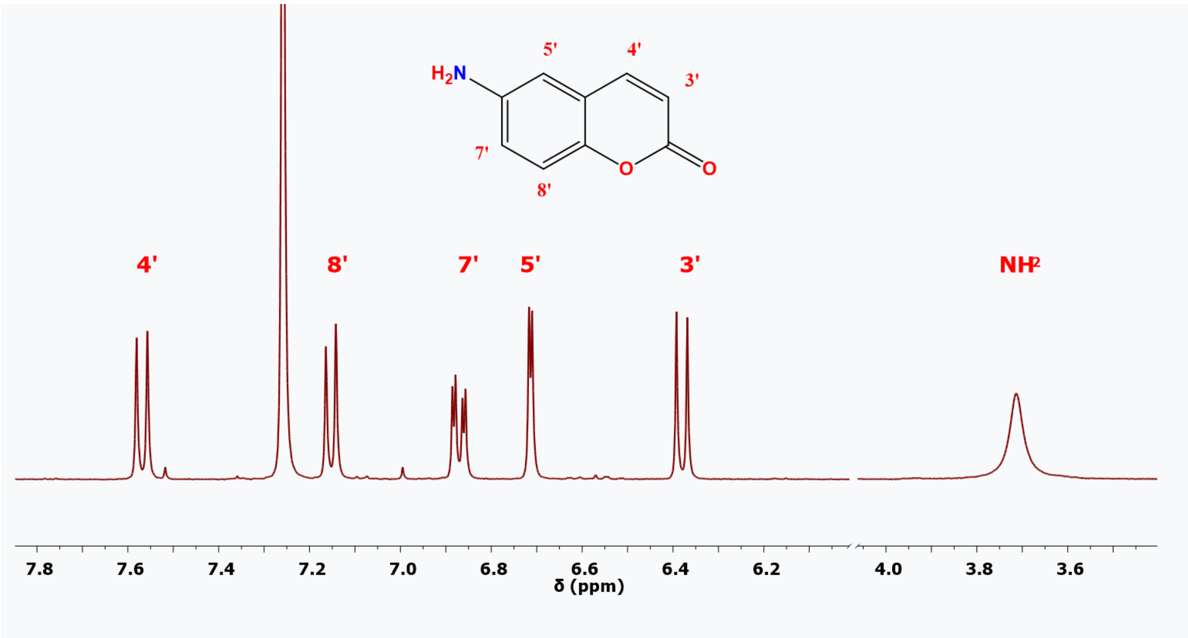

Figure S4. ^{1}H-NMR spectrum of **6'-aminocoumarin, N$_1$, in CDCl$_3$ at 300K**; where is observed the appearance of the signal corresponding to the amine proton. Insert: Structure with atom numbering for proton assignments.

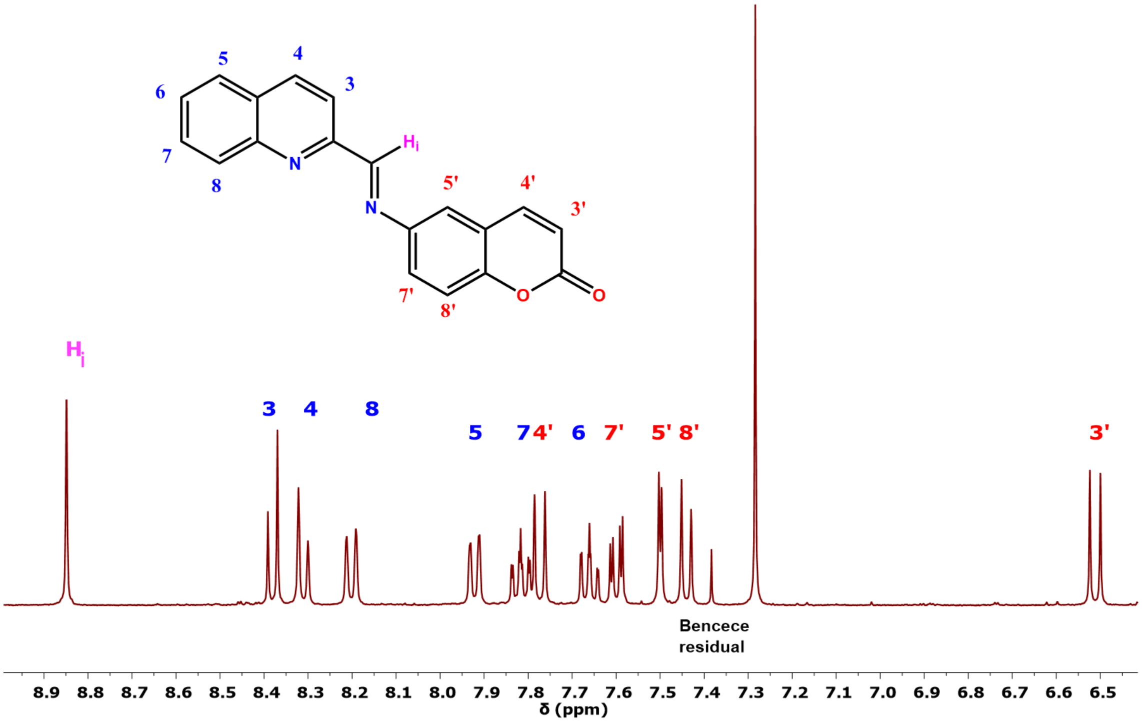

Figure S5. ^{1}H-NMR spectrum of **NN$_1$ in CDCl$_3$ at 300K**. Insert: Structure with atom numbering for proton assignments.

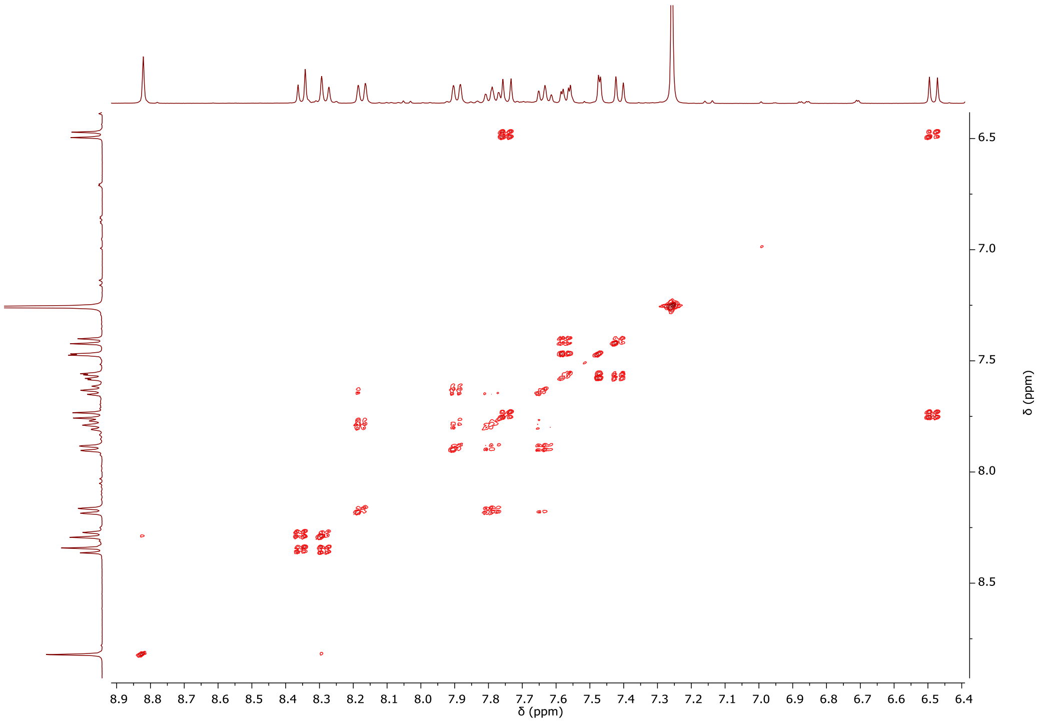

Figure S6. COSY NMR spectrum of **NN_1 in $CDCl_3$ at 300K.**

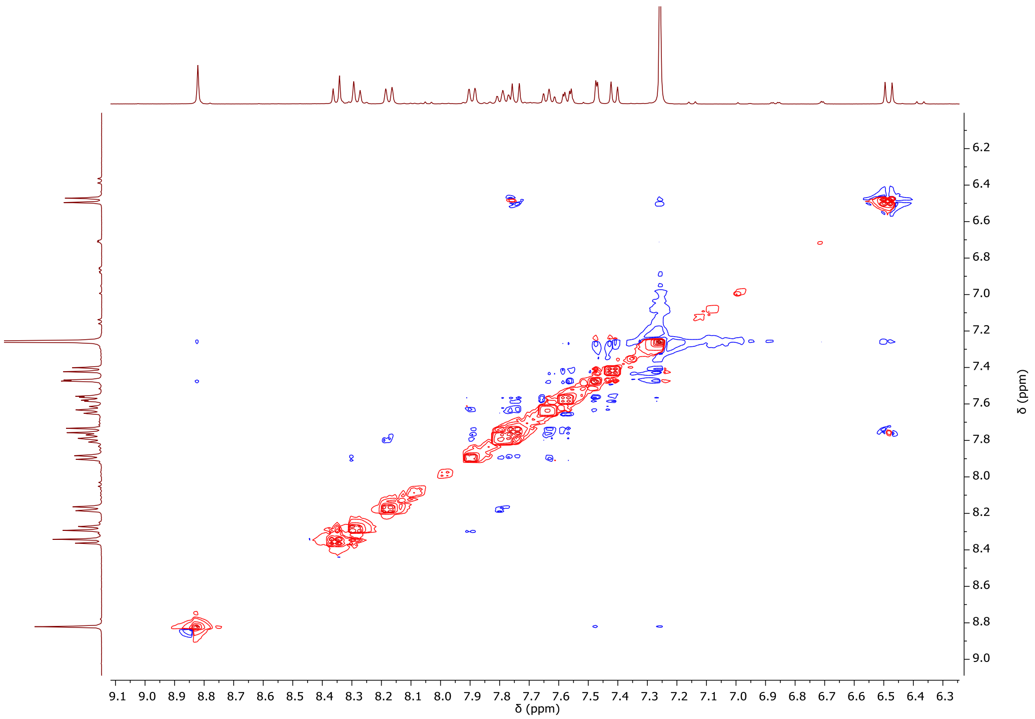

Figure S7. NOESY NMR spectrum of **NN_1 in $CDCl_3$ at 300K.**

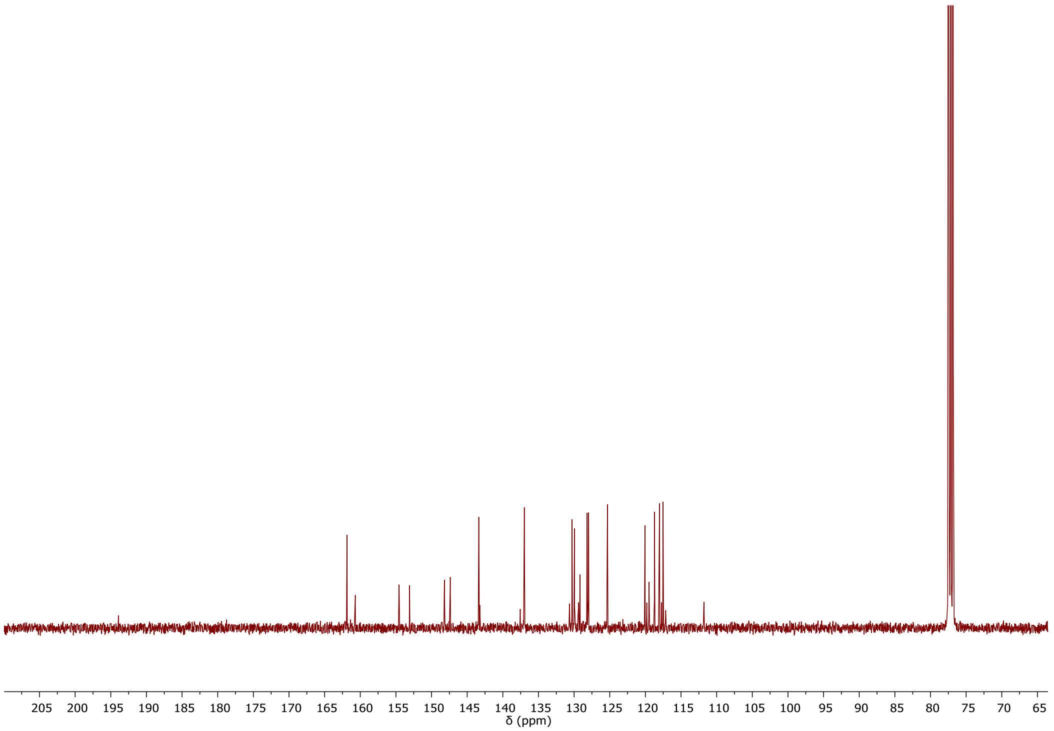

Figure S8. ^{13}C-NMR spectrum of **NN_1 in $CDCl_3$ at 300K.**

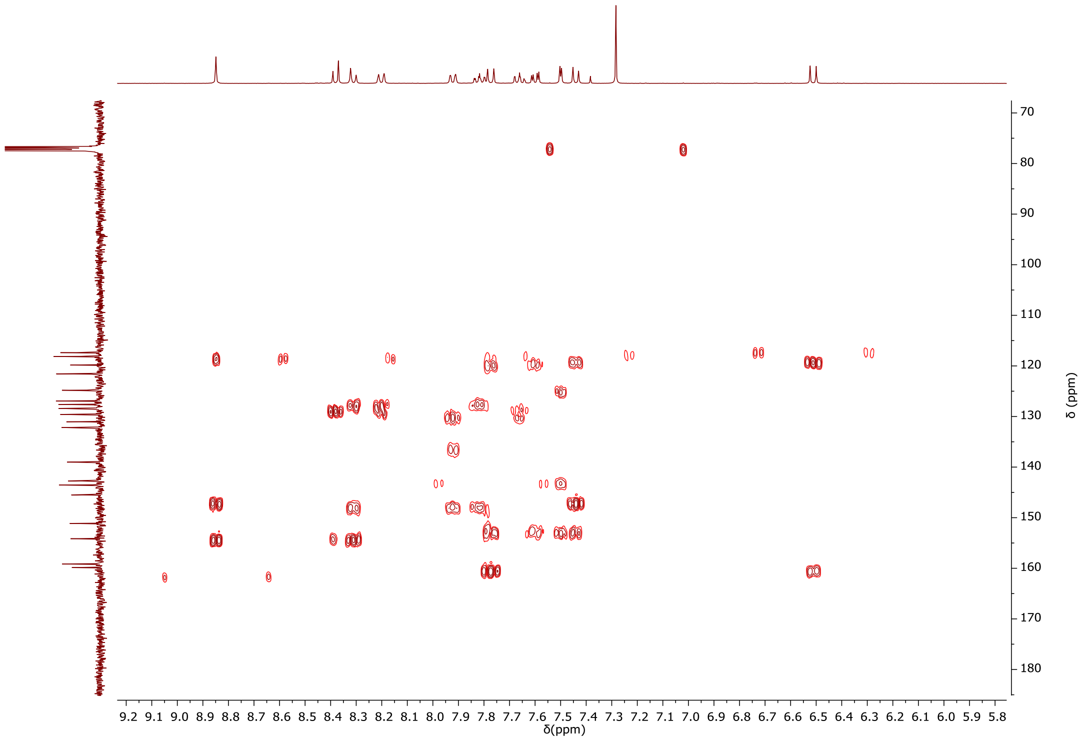

Figure S9. ^{1}H, ^{13}C HMBC NMR spectrum of **NN_1** in **$CDCl_3$ at 300K**.

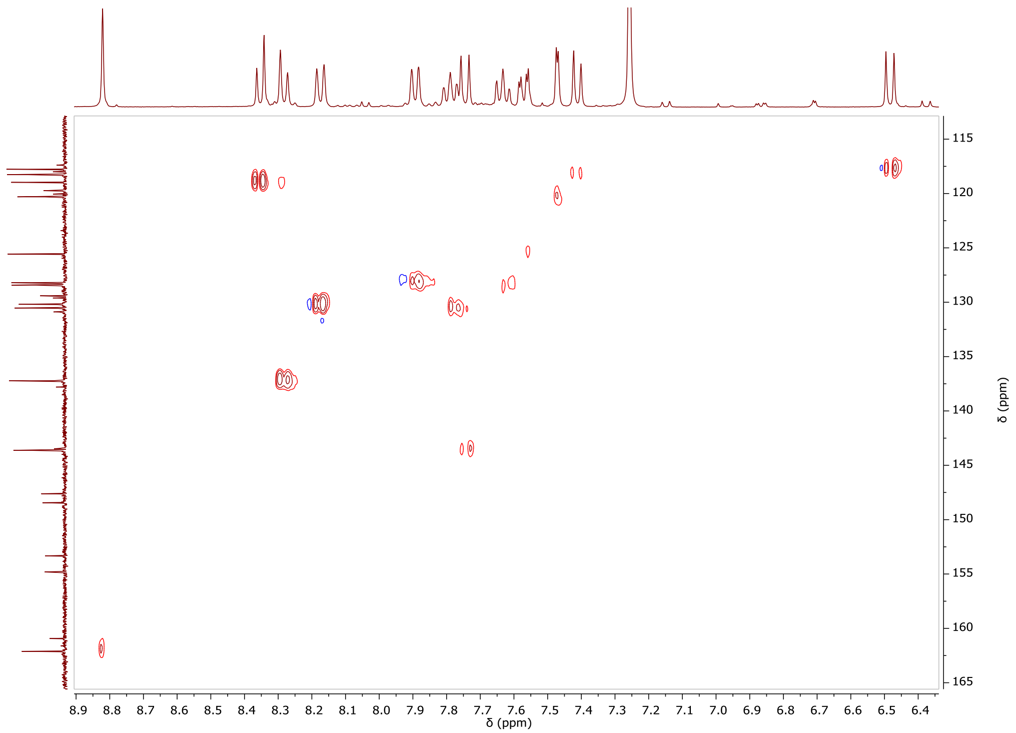

Figure S11. ^{1}H, ^{13}C HSQC NMR spectrum of **NN_1 in $CDCl_3$ at 300K.**

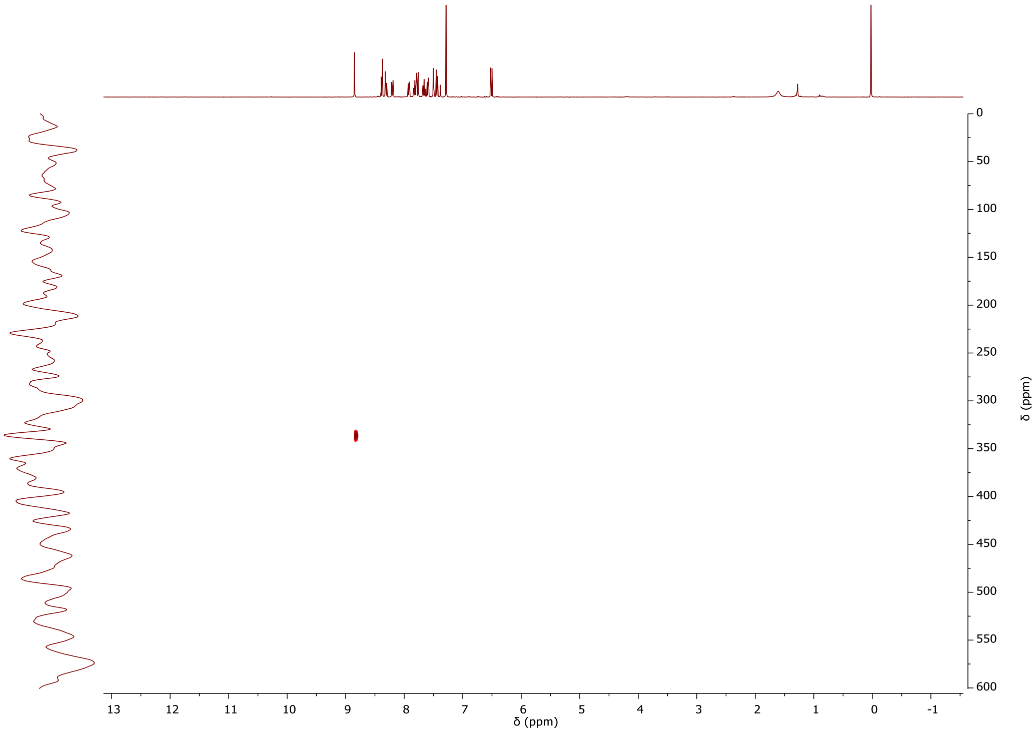

Figure S11. ^{1}H, ^{15}N HMBC NMR spectrum of **NN_1 in $CDCl_3$ at 300K.**

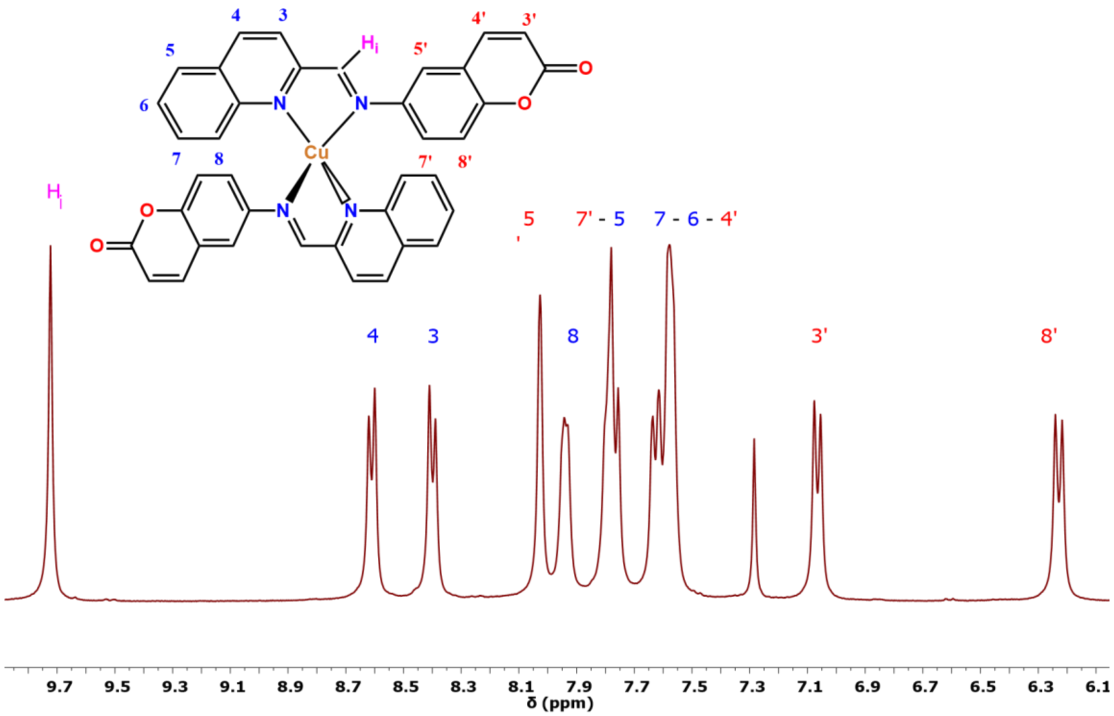

Figure S12. ^{1}H-NMR spectrum of **[Cu(NN$_1$)$_2$]ClO$_4$** in **CDCl$_3$ at 300K**. Insert: Structure with atom numbering for proton assignments.

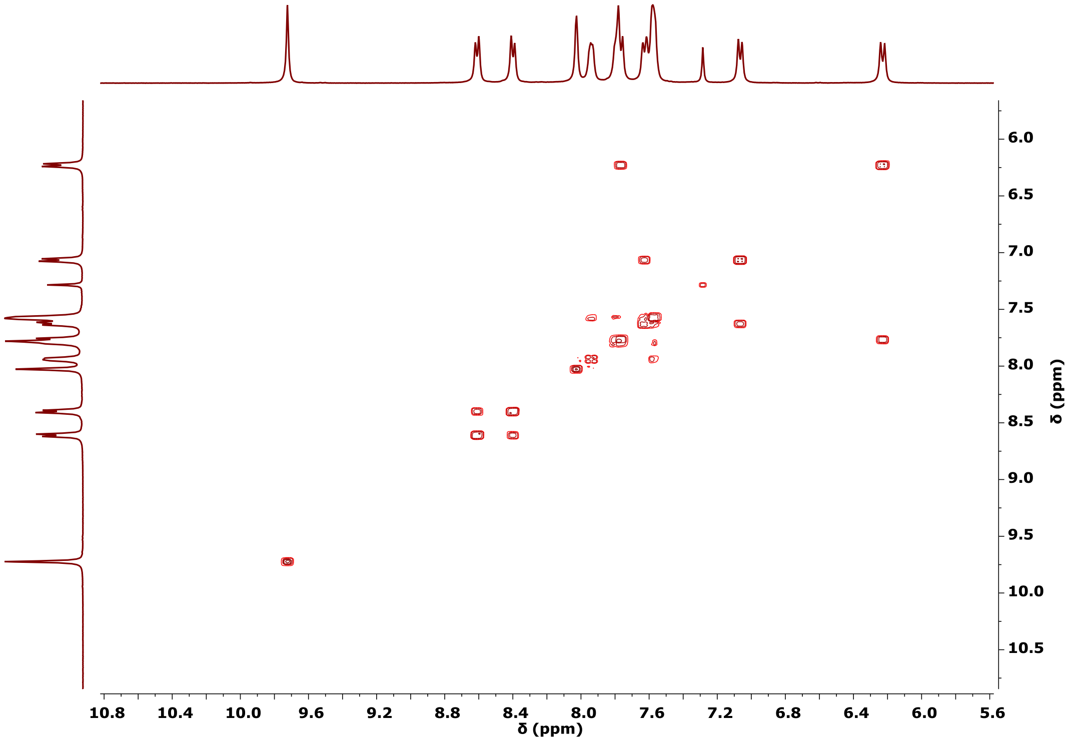

Figure S13. COSY NMR spectrum of **[Cu(NN$_1$)$_2$]ClO$_4$ in CDCl$_3$ at 300K.**

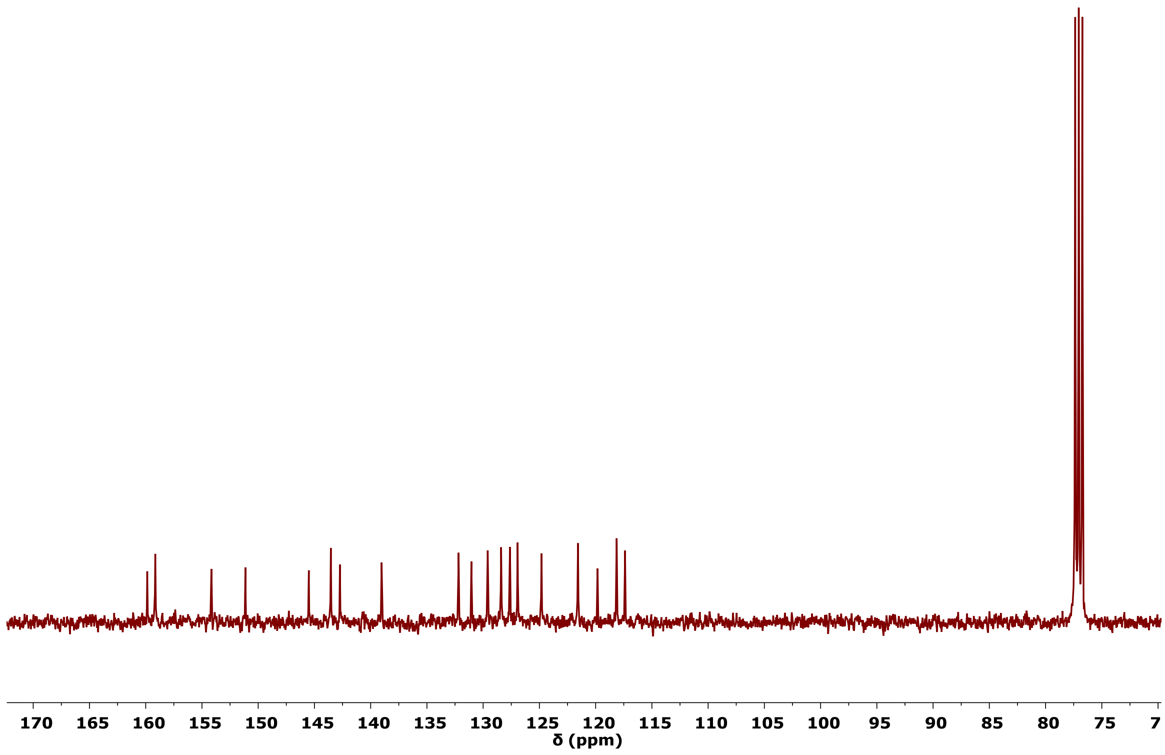

Figure S14. ^{13}C- NMR spectrum of **$[Cu(NN_1)_2]ClO_4$ in $CDCl_3$ at 300K.**

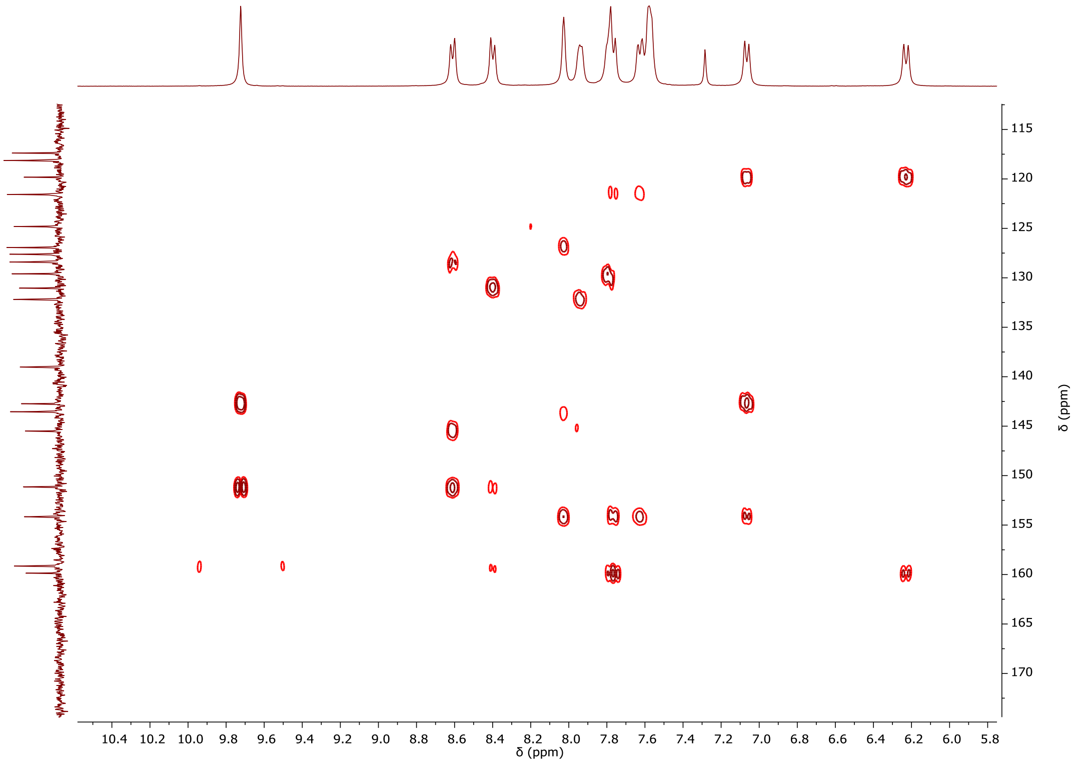

Figure S15. ^{1}H,^{13}C HMBC NMR spectrum of **$[Cu(NN_1)_2]ClO_4$ in $CDCl_3$ at 300K.**

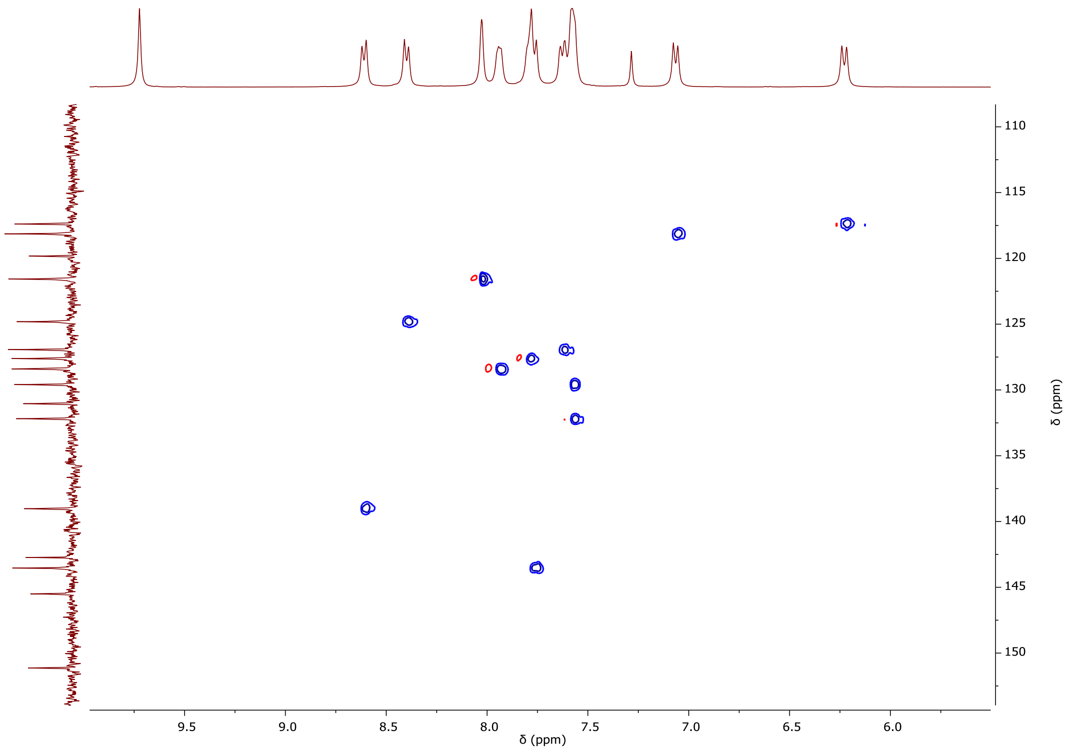

Figure S16. ^{1}H, ^{13}C HSQC NMR spectrum of **$[Cu(NN_1)_2]ClO_4$ in $CDCl_3$ at 300K.**

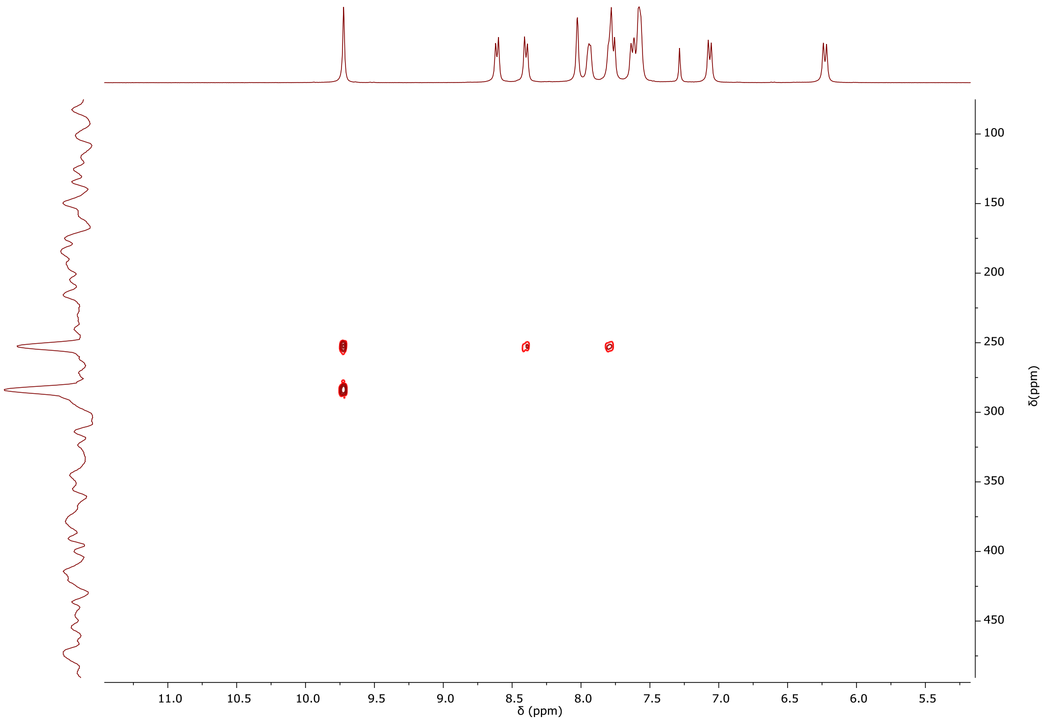

Figure S17. ^{1}H,^{15}N HMBC NMR spectrum of **$[Cu(NN_1)_2]ClO_4$ in $CDCl_3$ at 300K.**

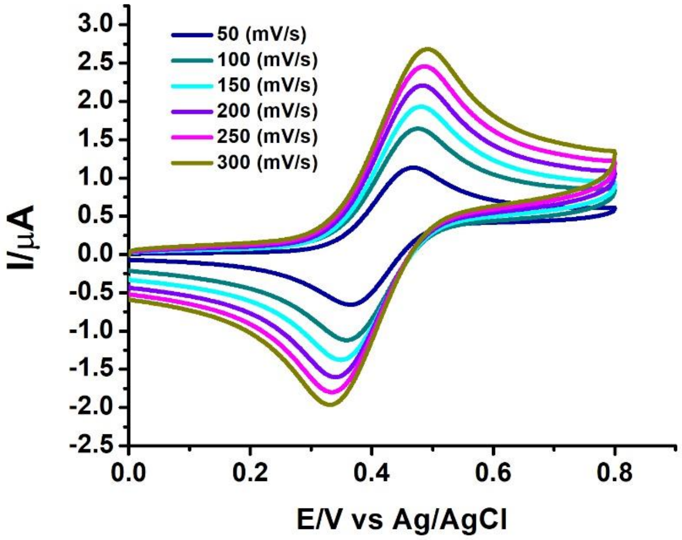

Figure S18. Cyclic voltammograms at several scan rate recorded for the oxidation of 2mmol/L, $[Cu(NN_1)_2]ClO_4$ complex in CH_2Cl_2, containing 0,1 mol/L TBAP.

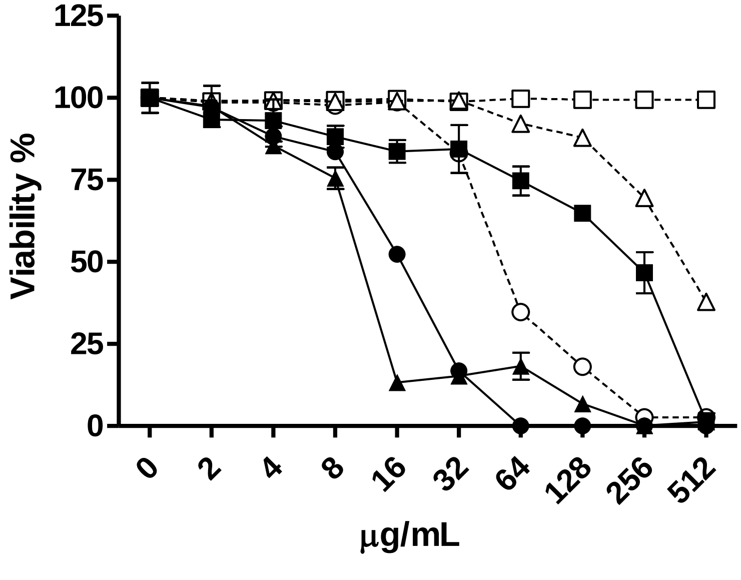

Figure S19. Viability of RT-GUT cells and *F. psychrophilum* 10094 (*F.s* 10094). The figure show the percentage of viable cells after treatment with copper (I) complex, coumarin, and copper salt, all normalized respect its control, (-○-) RT-GUT /copper (I) complex; (-□-) RT-GUT/coumarin; (-△-) RT-GUT/copper salt; (–●–) *F.s* 10094/copper (I) complex; (–■–) *F.s* 10094/coumarin; (–▲–) *F.s* 10094/copper salt.
